# Mindfulness and Athlete Burnout: A Systematic Review and Meta-Analysis

**DOI:** 10.3390/ijerph16030449

**Published:** 2019-02-03

**Authors:** Chunxiao Li, Yuxin Zhu, Mengge Zhang, Henrik Gustafsson, Tao Chen

**Affiliations:** 1 Faculty of Athletic Training, Guangzhou Sport University, Guangzhou 510500, China; 2 National Institute of Education, Nanyang Technological University, Singapore 637616, Singapore; 3 Department of Health and Physical Education, The Education University of Hong Kong, Hong Kong, China; s1114219@s.eduhk.hk (Y.Z.); mzhang@eduhk.hk (M.Z.); 4 Department of Pedagogical Studies, Karlstad University, SE-651 Karlstad, Sweden; henrik.gustafsson@kau.se; 5 Department of Coaching and Psychology, Norwegian School of Sport Sciences, 0086 Oslo, Norway

**Keywords:** meditation, stress, emotional exhaustion, sport, research synthesis

## Abstract

*Objective:* This review aims to identify, appraise, and synthesize studies reporting the relationship between mindfulness and athlete burnout and the effects of mindfulness-based interventions (MBIs) on athlete burnout. *Methods:* Studies were identified through searching six electronic databases using combinations of three groups of keywords and manual search. Two independent reviewers screened the searched studies, extracted data of the included studies, and assessed the study quality. The extracted data were synthesized qualitatively and quantitatively. *Results:* Ten studies consisting of two controlled trials, six surveys, and two interview studies met the inclusion criteria. The two controlled trials had weak methodological quality, and the remaining studies were of moderate to high research quality. Results of controlled trials and interview research generally showed that MBIs had positive effects in burnout prevention. Meta-analytic results indicated a negative association between mindfulness and burnout. *Conclusions:* There is some evidence showing that mindfulness was negatively associated with athlete burnout. However, given the small number of interventions and qualitative studies, there is limited evidence on whether MBIs are useful in preventing athlete burnout. More studies are needed to corroborate these findings.

## 1. Introduction

Even though sport participation can be enjoyable, athletes may also face numerous challenges, such as intensive training and heavy academic burden during the long period of athletic development [1,2]. Failing to meet these challenges might lead athletes to experience a maladaptive psychological syndrome known as athlete burnout [3]. Athlete burnout has been conceptualized as a multidimensional construct consisting of three dimensions: Emotional/physical exhaustion (i.e., feelings of psychosocial and physical fatigue), reduced sense of accomplishment (i.e., feelings of inefficacy and a trend to underestimate sports performance), and sport devaluation (i.e., negative feelings about the benefits of sports involvement) [4]. Based on the definition and conceptualization, the Athlete Burnout Questionnaire (ABQ) has been developed and widely used to measure the three burnout dimensions and global burnout (i.e., the composite score of the three burnout dimensions) [4].

The development of the ABQ has advanced the line of burnout research. Early research has shown that athlete burnout is associated with great distress, health problems, reduced sports performance, and termination of sports career [5,6]. It is therefore important to prevent and reduce athlete burnout. As athlete burnout can be caused by factors such as chronic stress, perfectionistic concerns, and decreased self-determined motivation, strategies such as cognitive behavior therapy, reducing perfectionistic mindset, and modifying organizational factors may be effective in the prevention and treatment of athlete burnout [7]. In addition, mindfulness techniques have been proposed as a potential strategy for combating athlete burnout [7].

Mindfulness is defined as “the awareness that emerges through paying attention, on purposes, and non-judgmentally to the unfolding of experience moment by moment” [8]. Indeed, mindfulness is an inherent capacity (dispositional mindfulness) that can be cultivated through mindfulness-based practice [9]. The characteristics of trait mindfulness or cultivated mindfulness-based skills (e.g., flexible, nondefensive, receptive, and present-focused awareness and attention) are believed to work together in bringing about beneficial outcomes [10]. For example, paying attention to the present moment rather than ruminating on the past or worrying about the future enables individuals to decrease emotional reactivity and enhance cognitive appraisal (e.g., observe a demand in a nonjudgmental manner).

Early review research found that dispositional mindfulness is negatively associated with occupational and academic burnout [11,12]. Systematic reviews of controlled trials also showed that mindfulness-based skills training or mindfulness-based interventions (MBIs) have positive effects on alleviating symptoms of stress and anxiety among healthy individuals and clinical populations [13,14]. Inspired by the growing empirical evidence and positive findings regarding the link between mindfulness and psychological outcomes in general psychology, mindfulness research in sport has received increasing attention over the past ten years [15]. For example, a number of studies revealed a beneficial effect of MBIs on athletic performance [16]. Recently, mindfulness has been proposed as a promising strategy to manage and prevent athlete burnout [7]. This is mainly based on the research from occupational settings, where promising results indicate the positive effect of mindfulness on mitigating stress and burnout symptoms [14]. 

In the sports context, several models have been proposed to explain athlete burnout [1], the most relevant to the present review being Smith’s cognitive–affective stress model, given that athlete burnout is mainly a stress-related symptom [17]. In Smith’s model, stress and burnout develop in parallel in a four-component framework, including situational, cognitive, physiological, and behavioral components. The first component, the situation, includes the interaction between the person and the environment (i.e., demands and resources). The second component is the cognitive appraisal of demands and recourses. The third component is the physiological response to the cognitive appraisal. If the demand is perceived as threatening, a physiological response arises to deal with the situation. The final component is the behavioral response, which includes various forms of coping, such as rigid inappropriate behaviors and withdrawal from the activity. All of these components are influenced by motivational and personality factors [17]. As mindfulness is a way to handle negative emotions and stress, it has been postulated that the mindful athlete would be less prone to burnout [18]. Initial research findings and theoretical assumptions suggest that burned-out athletes are more likely to ruminate and therefore tend to engage in maladaptive coping strategies [19,20]. Taken collectively, this provides a rationale for why mindfulness should be an important approach to decrease the risk of athlete burnout.

Although related empirical studies about mindfulness and athlete burnout are available [19,21], no attempt has been made to systematically synthesize and quantify the findings. We therefore conducted a systematic review and meta-analysis to fill this gap, provide evidence-based practice in burnout prevention, and inform future research in this area. Specifically, we aimed to identify, appraise, and synthesize studies examining the relationship between mindfulness and athlete burnout and/or the effects of MBIs on athlete burnout.

## 2. Materials and Methods

### 2.1. Protocol and Registration

This systematic review was conducted by following the preferred reporting items for systematic reviews and meta-analyses guideline [22], which is an evidence-based approach for reporting in systematic reviews and meta-analyses. 

### 2.2. Eligibility Criteria

Studies included in this review must meet the following criteria: (i) Participants should be athletes (i.e., a person who is regularly involved in sports training); (ii) they must be empirical studies examining either the relationship between mindfulness and burnout or the effect of MBIs on burnout; and (iii) the relationship between mindfulness and burnout or the effect of MBIs on burnout is reported. Studies were excluded if they (i) were not original/empirical studies, such as book review and commentary; (ii) did not recruit athletes as participants; (iii) did not examine the association between mindfulness and athlete burnout; or (iv) did not investigate the effects of MBIs on athlete burnout.

### 2.3. Information Sources, Search, and Study Selection

One of the authors (YZ) conducted the literature search using six major electronic databases (i.e., CINAHL Plus, MEDLINE, PsycINFO, Scopus, SPORTDiscus, and Web of Science) on 22 December 2018. Three groups of keywords, identified based on our research expertise and early mindfulness/burnout research, were combined for the search: (i) “athlete*” OR “sport*”; AND (ii) “mindfulness” OR “meditat*” OR “contemplative science” OR “MBSR” OR “MBCT” OR “acceptance and commitment therapy” OR “ACT” OR “psychological flexibility”; AND (iii) “burnout” OR “exhaustion” OR “overtraining” OR “dropout” OR “staleness” OR “depersonalization” OR “devaluation”. Additional articles were identified by reading through the reference list of the included studies and relevant review articles. We also wrote to experts in the field to ascertain whether they knew additional articles on our review topic. The searched articles were exported into EndnoteX8 to identify duplicates. After removing the duplicates, two reviewers (YZ and MZ) independently screened the remaining articles via titles and abstracts. Full texts were then sought if the titles and abstracts provided insufficient information for determining the eligibility of articles. Disagreements on article selection were resolved by consulting a third reviewer (CL).

### 2.4. Data Items and Collection Process

The following data items were extracted from the included studies: (i) Study design (e.g., cross-sectional survey and quasi-experiment), (ii) participant characteristics (e.g., sample size and age), (iii) training program of MBI if applicable (e.g., content and dose), (iv) outcome measures (e.g., mindfulness and burnout), (v) key findings, and (vi) effect sizes or quantitative information for effect size calculation. We also wrote to authors of the included studies to obtain the raw data for effect size calculation if applicable. Two reviewers (YZ and MZ) extracted the data independently. Several discrepancies were resolved after consulting a third reviewer (CL).

### 2.5. Risk of Bias in Individual Studies

As each critical appraisal tool is usually designed for assessing a specific type of research, different tools were used for the purpose of our research. Specifically, the methodological quality of cross-sectional surveys, qualitative studies, and controlled trials was appraised by the adapted 10-item AXIS tool [23,24], the 10-item Joanna Briggs Institute (JBI) critical appraisal tool [25], and the modified 27-item Downs and Black checklist [26], respectively. The overall score of the adapted AXIS and JBI ranged from 0 to 10 and a total score of 0–4, 5–7, and 8–10 represents low, moderate, and high methodological quality, respectively [24,27]. The total score of the Downs and Block checklist ranges from 0 to 28 with a higher percentage of the maximum score representing better methodological quality: <50% (weak), 50%–69% (fair), 70%–79% (good), and 80%–100% (very good) [28]. Two reviewers (Y.Z. and M.Z.) assessed the methodological quality of the included studies. Disagreements were resolved after discussion with a third reviewer (CL).

### 2.6. Summary Measures and Synthesis of Results

Extracted data (e.g., sample size and outcome measure) across studies were summarized and synthesized in narrative text. Although researchers can conduct a meta-analysis with only two independent effect sizes, the findings of the two included intervention studies were only qualitatively synthesized to avoid type I/II errors [29]. We also qualitatively summarized the results of the two included qualitative studies. Meta-analyses were used to combine the effect sizes (i.e., *r*) across individual studies where the relationship between mindfulness and athlete burnout was reported. A random-effects model was applied to generate the study findings [30]. The *Q* test was used to assess heterogeneity of effect sizes with a significant *Q* value indicating heterogeneity (i.e., the true effect size varies across studies). *I^2^* statistics were employed to evaluate the magnitude of heterogeneity, with an *I^2^* value higher than 25%, 50%, and 75% indicating low, moderate, and high heterogeneity, respectively [31].

### 2.7. Risk of Bias across Studies and Additional Analysis

Publication bias was addressed using the analysis of “fail-safe number” for effect size [32,33]. This analysis determines the number of nonsignificant missing studies needed to bring an aggregated correlation coefficient to a small effect (*r* = 0.10). If the number of missing studies is relatively large compared with the number of studies included in a meta-analysis, it is unlikely that there is publication bias. Moderation analysis was not conducted in this study because only a small number of the studies was included in this review, increasing the second-order sampling errors [34]. All the meta-analyses were conducted in Comprehensive Meta-Analysis version 2.0 (Biostat, Englewood, NJ, USA).

## 3. Results

This section may be divided by subheadings. It should provide a concise and precise description of the experimental results, their interpretation, as well as the experimental conclusions that can be drawn.

### 3.1. Study Selection

**Figure 1:**
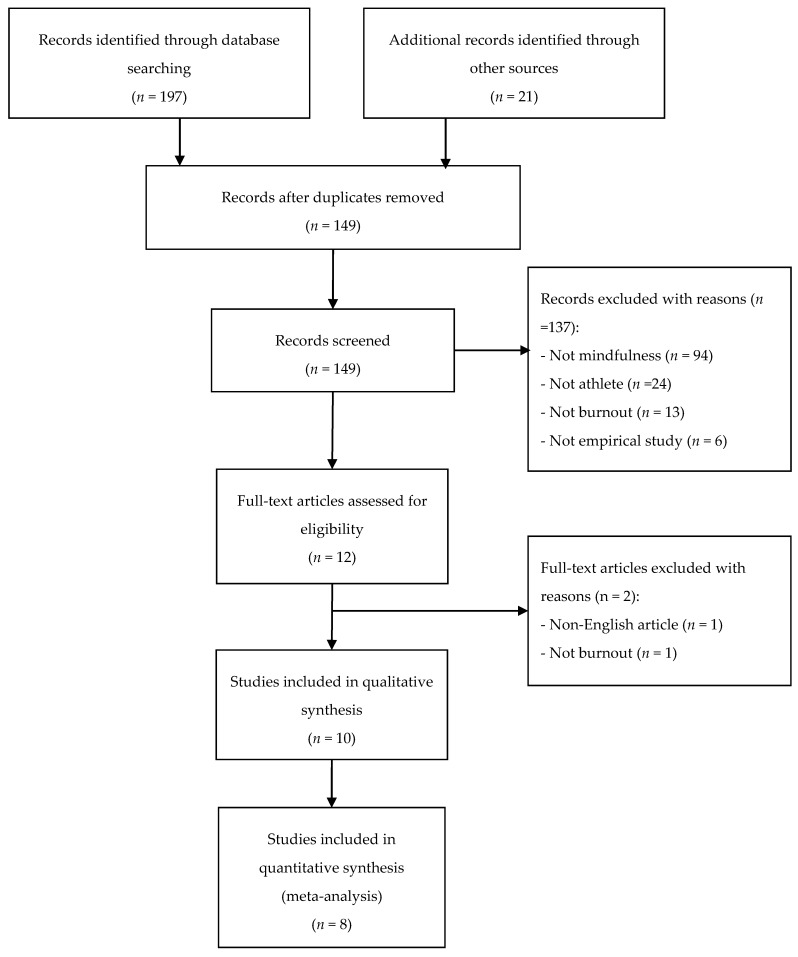
Study selection process.

The literature search retrieved 218 studies. After removing the duplicates, 149 studies were screened using titles and abstracts. A total of 137 publications were excluded and the remaining 12 studies were further reviewed by reading through full texts. Finally, ten studies meeting the inclusion criteria were included, with eight of these reporting the relationship between mindfulness and burnout being compatible for meta-analysis. Details of the study selection process are presented in Figure 1.

### 3.2. Study Characteristics

A summary of selected studies is presented in Table 1. These studies included two controlled trials (#1 and #2), five cross-sectional surveys (#3–7, one prospective survey (#8), and two qualitative studies (#9 and #10), published from 2013 to 2019 and carried out in six different countries. Three studies were conducted in Norway (#1, #2, and #6), two in Sweden (#3 and #10), two in China (#4 and #7), one in Japan (#8), one in South Africa (#5), and one in India (#9). The sample size ranged from 1 to 385 and participants’ mean age ranged from 15.44 to 19.84 years across the included studies. The percentage of gender was generally balanced (range 40%–67% except for study #10, in which a female athlete was recruited).

Three different kinds of MBIs were found in the included studies. Two studies (#1 and #9) adopted the same program that required athletes to do a sitting mediation and body scanning session (10–30 min/session) every day for 12 weeks. Study #2 also used a 12-week intervention program but with different training contents, including both attention training (≥5 times/week, 12 min/session) and reflection (every third week/session, 120–150 min/session) sessions. Finally, a case study (#10) applied a 20-week active coaching program that combined a mindfulness schema (three separate normal days), body scanning (≥2 times/day, 20–40 min/session), and Qigong exercises (≥2 times/day, 20–60 min/session).

The measurement tools used for mindfulness and athlete burnout were basically consistent across the included studies. The Mindful Attention Awareness Scale (MAAS) [35] was popularly used in five studies (#1–4 and #6), whereas the Athlete Mindfulness Questionnaire [36] was employed in studies #7 and #8, and the Freiburg Mindfulness Inventory [37] in study #5. The remaining two studies (#9 and #10) did not assess participants’ mindfulness level. For burnout, the ABQ [4] was the most popular tool employed by eight studies (#1–7 and #10). Study #8 used the Burnout Scale for University Athletes [38], while study #9 did not measure participants’ burnout level.

### 3.3. Risk of Bias

The results of quality appraisal showed that both included controlled trials (#1 and #2) only scored 32% of the total score, indicating low methodological quality. The six surveys (#3–8) and two qualitative studies (#9 and #10) had moderate to high methodological quality (range 7–9). The detailed results of quality appraisal are presented in Supplementary Tables S1–S3.

### 3.4. Results of Individual Studies

#### 3.4.1. Controlled Trials

A nonrandomized controlled trial (#1) examined the effectiveness of a 12-week mindfulness program that combined attention training (≥5 times/week, 12 min/session) and reflection (every third week/session, 120–150 min/session) sessions on athlete burnout among youth athletes from six different sports. Compared with the blanket control group (*n* = 51, 19 dropouts), the intervention group participants (*n* = 27, 2 dropouts) reported a significant decrease of global burnout (partial *η^2^* = 0.11) and reduced sense of accomplishment (partial *η^2^* = 0.15), but not for emotional/physical exhaustion (partial *η^2^* = 0.04) and sport devaluation (partial *η^2^* = 0.02).

In another nonrandomized controlled trial (#2), 27 out of 29 youth athletes from five different sports completed an MBI program (12-week sitting meditation and body scanning, 3–7 times/week, 10–30 min/session). Compared with the blank control group (*n* = 48 with 27 attending post-test), the intervention group had a significantly lower level of global burnout (partial *η^2^* = 0.24), reduced sense of accomplishment (partial *η^2^* = 0.28), and sport devaluation (partial *η^2^* = 0.13) post-test. However, emotional/physical exhaustion was not significantly decreased after the intervention (partial *η^2^* = 0.08).

#### 3.4.2. Survey

Despite varying participant characteristics (i.e., sample size, percentage of gender, mean age, and sports), results of the five cross-sectional surveys (#3–7) and one prospective survey (#8) generally indicated that mindfulness was negatively related to global burnout and the three burnout dimensions (*r* = −0.14 to −0.55).

#### 3.4.3. Qualitative Research

Study #9 followed the same intervention program used in study #2. The effectiveness of the intervention was examined through interviewing six youth athletes from three sports. The interview results indicated that the intervention program had a significantly positive effect on reducing athlete burnout.

A case study (#10) examined the effect of a 20-week active coaching program on athlete burnout on a female shooter who had demonstrated burnout symptoms for six months. The participant was instructed to practice a mindfulness schema (three separate normal days), body scanning (≥2 times/day, 20–40 min/session), and Qigong exercises (≥2 times/day, 20–60 min/session). The results showed that mindfulness and Qigong techniques were useful in the recovery from athlete burnout.

### 3.5. Synthesis of Results and Risk of Bias across Studies

Table 2 shows the results of meta-analysis. As expected, mindfulness had a significantly negative association with global burnout and its three dimensions (*r* = −0.28 to −0.42). Among the three burnout dimensions, mindfulness had the strongest association with emotional/physical exhaustion. Meanwhile, we detected a low risk of publication bias (*k* = 4–7; fail-safe number = 13–14).

## 4. Discussion

The current systematic review and meta-analysis provided a general result regarding the relationship between mindfulness and athlete burnout, and the effects of MBIs on athlete burnout. Despite the relatively small number of studies included, our meta-analytic results indicated that there is some evidence supporting a negative association between mindfulness and athlete burnout. Furthermore, mindfulness had the strongest association with emotional/physical exhaustion when compared with the other two burnout dimensions.

Despite the strongest association, the results of the two controlled trials (#1 and #2) showed that athletes’ emotional/physical exhaustion was not improved after undergoing MBIs. Given that emotional/physical exhaustion (i.e., the core dimension of athlete burnout) was not significantly improved, it might be difficult to ascertain whether MBIs were effective in burnout prevention. This finding may be attributed to the relatively short intervention period (i.e., 12 weeks) as emotional/physical exhaustion is considered as a chronic stress-related dimension, which is relatively stable within a short period of time [44]. This is evidenced by the included case study (#10), in which an athlete successfully recovered from the burnout symptoms after receiving 20 weeks of MBIs. Worthy of caution is that the case study involved a burned-out athlete whereas the two controlled trials did not. Furthermore, the controlled trials and case study used different intervention components. These differences may affect the reported effectiveness of MBIs on emotional/physical exhaustion.

The results of intervention and qualitative research generally suggested that MBIs might be effective in reducing levels of global burnout and reduced sense of accomplishment. One possible explanation for these positive findings is that mindfulness can help athletes to keep an open and nonjudgmental orientation toward their experiences in the present moment [35]. Therefore, it might be possible that athletes who have a high level of mindfulness are less likely to suffer from emotional distress and reduced satisfaction derived from critical self-evaluation or repetitive thought processes.

According to the existing empirical studies, a few variables may account for the relationship between mindfulness and athlete burnout. Gustafsson et al. [18] found that mindfulness had an indirect effect on athlete burnout through negative/positive affect. As negative affect has been viewed as an indicator of burnout susceptibility [45], mindfulness is likely to prevent burnout through adjusting athletes’ affect. Zhang and colleagues [40] proposed another possible mechanism. They claimed that athletes with high levels of mindfulness tend to have low levels of experiential avoidance (i.e., willingness to avoid negative experience because of the distress brought on by the experience [46], which may in turn minimize the maladaptive influences of stressors and other negative factors on burnout. Given that only a small number of studies have examined these possible mechanisms, further investigations are needed to confirm them.

Some other literature gaps were also identified through the present review. Future research should recruit participants from different countries or cultural backgrounds, as samples involved in our review were limited to only a few geographical locations. Additionally, findings in this review are more adaptive to young athletes given that the majority of participants were adolescents. This is because the stressors between adolescent and adult athletes are different (e.g., adolescent athletes may face academic challenges, while adult athletes may not) [47]. Thus, future studies may consider recruiting adult athletes. Moreover, the participants (except for the case study) were generally healthy athletes, rather than those who are identified with (severe) burnout symptoms. Therefore, it is necessary to recruit athletes with high burnout levels in future research to advance our understanding of the effects of MBIs on athlete burnout.

As only a small number of intervention studies with low research quality was included in this review, more (randomized) controlled trials with high research quality are needed to replicate and extend our findings. It is worth noting that none of the included intervention studies reported adverse events. As MBIs are offered worldwide, some adverse events have been reported in early research. For example, Kabat-Zinn and Chapman-Waldrop [48] noted that patients with chronic pain were less likely to complete MBIs in comparison with those with stress-related disorders. To facilitate transparent reporting and avoid reporting biases, we suggest that future studies report adverse events.

Regarding the outcome measures used across the included studies, five of the studies used the MAAS [35]. Although the MAAS is a popular and unidimensional tool for measuring dispositional mindfulness, there is a trend toward measuring multiple facets of mindfulness [49]. For example, the Five Facet Mindfulness Questionnaire [50] has strong psychometrics that can be applied in future research [51]. It would be interesting to know which facets of mindfulness have a closer association with athlete burnout. As expected, most of the included studies adopted the same tool (i.e., ABQ) to assess athlete burnout [4]. As most of the participants involved in this review are student athletes, the burnout measure should involve both sport and school burnout because they are constantly intertwined. Therefore, future studies may consider using other measures to evaluate relevant burnout dimensions (e.g., Sport Burnout Inventory—Dual Career Form [52]).

Despite the encouraging and intriguing findings presented, the present review is subject to three major limitations. First, only English peer-reviewed journal articles were included, precluding some potentially relevant studies published in other languages. Second, moderate heterogeneity was found in two of the meta-analytic results. As only a relatively small number of studies was included in our meta-analyses, it could be problematic to explore the causes of heterogeneity. However, the process may be problematic anyway given the many differences across studies (e.g., study quality and participant characteristics) that one can purposely select to explain the causes of heterogeneity [53]. Finally, only ten articles with different methodological approaches and quality were included in this review, so it is difficult to draw a definitive conclusion. However, some important research gaps that we believe will inform future research directions were identified through our review.

## 5. Conclusions

There is some evidence supporting the negative association between dispositional mindfulness and athlete burnout among young athletes. However, given the small number of interventions and qualitative studies, it is still unclear whether MBI is an effective approach for burnout prevention. Intervention studies with rigorous methodology are warranted to test the efficiency of different MBIs on athlete burnout. Future research should also investigate potential mechanisms underlying the relationship between mindfulness and athlete burnout as well as some other research gaps identified herein.

## Figures and Tables

**Table 1:** Characteristics of the included studies.

Study #	First Author (Year), Country	Participants	Intervention	Measures	Key findings	Quality
*Controlled trial*						
1	Moen (2016), Norway [39]	*n* = 78, 67% Female, *M*_age_ = 18.50 (range = NA), from 6 different sports; Intervention group: *n* = 27, 2 dropout; Control group: *n* = 51, 19 dropout	Intervention: (12-week attention training, ≥ 5 times/week, 12 min/session) + (reflection sessions, every third week/ session, 120–150min/session) Control: No treatment	MAAS, ABQ	Significant intervention effects on decreasing burnout levels	Low
2	Moen (2015), Norway [21]	*n* = 77, 51% Female, *M*age = 18.50 (range = 16–20), from 5 different sports; Intervention group: *n* = 29, 6 dropout; Control group: *n* = 48, 21 dropout	Intervention: 12-week sitting meditation and body scanning, 3–7 times/week, 10–30 min/session Control: No treatment	MAAS, ABQ	Significant intervention effects on decreasing burnout levels	Low
*Survey*						
3	Gustafsson (2015), Sweden [18]	*n* = 233, 57% Female, *M*_age_ = 17.50 (range = 15–19), from 4 different sports	NA	MAAS, ABQ	Mindfulness was negatively related to the three burnout dimensions	High
4	Zhang (2016), China [40]	*n* = 385, 43% Female, *M*_age_ = 15.44 (range = 12–18), from 21 different sports	NA	MAAS, ABQ	Mindfulness was negatively related to the three burnout dimensions	High
5	Walker (2013), South Africa [41]	*n* = 104, 50% Female, *M*_age_ = 16.00, (range = 14–19), tennis	NA	FMI, ABQ	Mindfulness was negatively related to global burnout and its three dimensions	High
6	Moen (2015), Norway [42]	*n* = 382, 45% Female, *M*_age_ = 18.50 (range = 17–20), from 10 different sports	NA	MAAS, ABQ	Mindfulness was negatively related to reduced sense of accomplishment and sport devaluation	Moderate
7	Zhang (2017)—Study 5, China [36]	*n* = 379, 43% Female, *M*_age_ = 19.59 (range = 16–35), from 20 different sports	NA	AMQ, ABQ	Mindfulness was negatively related to the three burnout dimensions	High
8	Amemiya (2019), Japan [38]	*n* = 124, 40% Female, *M*_age_ = 19.84 (range = NA), from 6 different sports	NA	AMQ, BOSA	Mindfulness was negatively related to global burnout	High
*Qualitative research*						
9	Furrer (2015), India [43]	*n* = 6, 50% Female, *M*_age_ = 18.50 (range = 18–20), from 3 different sports	Intervention: 12-week sitting meditation and body scanning, 3–7 times/week, 10–30 min/session	Semi-structured interview	Mindfulness training had a positive effect on burnout prevention	Moderate
10	Jouper (2013), Sweden [19]	*n* = 1, 100% Female, unknown age, elite shooter with burnout symptoms	Intervention: 20-week active coaching, including (mindfulness schema, 3 separate normal days) + (body scanning, ≥ 2 times/day, 20–40 min/session) + (emotional qigong, ≥ 2 times/day, 20–60 min/session)	ABQ, meeting feedback	Mindfulness and Qigong techniques helped the case to recover from burnout	High

MAAS = Mindfulness attention awareness scale; ABQ = Athlete burnout questionnaire; NA = Not applicable; FMI = Freiburg mindfulness inventory; AMQ = Athlete mindfulness questionnaire; BOSA = Burnout scale for university athletes.

**Table 2:** Meta-analytic results of the relationship between mindfulness and burnout.

Variable	Study #	*k*	*n*	*r*	95% CI	Q (df)	*I^2^*	Fail-Safe *n*
Mindfulness and global burnout	#1, #2, #5, and #8	4	383	–0.42 **	(–0.50, –0.33)	1.47 (3)	0.00	14
Mindfulness and reduced sense of accomplishment	#1, #2, #3, #4, #5, #6, and #7	7	1638	–0.29 **	(–0.33, –0.24)	3.57 (6)	0.00	14
Mindfulness and emotional/physical exhaustion	#1, #2, #3, #4, #5, and #7	6	1256	–0.33 **	(–0.42, –0.24)	13.82 (5) *	63.82	13
Mindfulness and sport devaluation	#1, #2, #3, #4, #5, #6, and #7	7	1638	–0.28 **	(–0.35, –0.21)	14.35 (6) *	58.19	14

** *p* < 0.01, * *p* < 0.05; study # is identical to those presented in Table 1; *k* = Number of independence samples; *r* = Average-weighted correlation coefficient; CI = Confidence interval.

## Supplementary Materials

The following are available online at http://www.mdpi.com/1660-4601/16/3/449/s1, Table S1: Trials, Table S2: Survey, Table S3: Qualitative Research.
