# Supplementary material for: Mindfulness and Athlete Burnout: A Systematic Review and Meta-Analysis
Source: Int J Environ Res Public Health. 2019 Feb 3;16(3):449. doi: 10.3390/ijerph16030449 (PMC6388258; doi:10.3390/ijerph16030449)
Supplement: Supplementary file 1 [file ijerph-16-00449-s001.pdf]

Review

Mindfulness and Athlete Burnout: A Systematic Review and Meta-Analysis

Chunxiao Li, Yuxin Zhu, Mengge Zhang, Henrik Gustafsson and Tao Chen

Table S1. Trials.

Study #	First author (year)	Q1	Q2	Q3	Q4	Q5	Q6	Q7	Q8	Q9	Q10	Q11	Q12	Q13	Q14	Q15	Q16	Q17	Q18	Q19	Q20	Q21	Q22	Q23	Q24	Q25	Q26	Q27	Total (%)	Qualit y
1	Moen (2016)	1	0	0	1	0	1	0	0	0	1	0	0	1	0	0	0	0	1	0	1	1	1	0	0	0	0	0	9 (32%)	Weak
2	Moen (2015)	1	0	0	1	0	1	0	0	0	1	0	0	1	0	0	0	0	1	0	1	1	1	0	0	0	0	0	9 (32%)	Weak

Note. 1 = Yes, 0 = No/Unable to determine (except Q5: 2 = Yes, 1 = Partially, 0 = No)

Q1: Is the hypothesis/aim/objective of the study clearly described?

Q2: Are the main outcomes to be measured clearly described in the Introduction or Methods section?

Q3: Are the characteristics of the patients included in the study clearly described?

Q4: Are the interventions of interest clearly described?

Q5: Are the distributions of principal confounders in each group of subjects to be compared clearly described?

Q6: Are the main findings of the study clearly described?

Q7: Does the study provide estimates of the random variability in the data for the main outcomes?

Q8: Have all important adverse events that may be a consequence of the intervention been reported?

Q9: Have the characteristics of patients lost to follow-up been described?

Q10: Have actual probability values been reported (e.g. 0.035 rather than <0.05) for the main outcomes except where the probability value is less than 0.001?

Q11: Were the subjects asked to participate in the study representative of the entire population from which they were recruited?

Q12: Were those subjects who were prepared to participate representative of the entire population from which they were recruited?

Q13: Were the staff, places, and facilities where the patients were treated, representative of the treatment the majority of patients receive?

Q14: Was an attempt made to blind study subjects to the intervention they have received?

Q15: Was an attempt made to blind those measuring the main outcomes of the intervention?

Q16: If any of the results of the study were based on "data dredging", was this made clear?

Q17: In trials and cohort studies, do the analyses adjust for different lengths of follow-up of patients, or in case-control studies, is the time period between the intervention and outcome the same for cases and controls?

Q18: Were the statistical tests used to assess the main outcomes appropriate?

Q19: Was compliance with the intervention/s reliable?

Q20: Were the main outcome measures used accurate (valid and reliable)?

Q21: Were the patients in divergent intervention groups (trials and cohort studies) or were the cases and controls (case-control studies) recruited from the same population?

Q22: Were study subjects in divergent intervention groups (trials and cohort studies) or were the cases and controls (case-control studies) recruited over the same period of time?

Q23: Were study subjects randomized to intervention groups?

Q24: Was the randomized intervention assignment concealed from both patients and health care staff until recruitment was complete and irrevocable?

Q25: Was there adequate adjustment for confounding in the analyses from which the main findings were drawn?

Q26: Were losses of patients to follow-up taken into account?

Q27: Did the study have sufficient power to detect a clinically important effect where the probability value for a difference being due to chance is less than 5%?

Table S2. Survey.

**Study #**	**First author (year)**	**Q1**	**Q2**	**Q3**	**Q4**	**Q5**	**Q6**	**Q7**	**Q8**	**Q9**	**Q10**	**Total**	**Quality**
3	Gusstafsson (2015)	1	1	0	1	1	1	1	1	1	1	9	High
4	Zhang (2016)	1	1	0	1	1	1	1	1	1	1	9	High
5	Walker (2013)	1	1	0	1	1	1	1	1	1	1	9	High
6	Moen (2015)	1	1	0	1	1	1	1	1	0	0	7	Moderate

7	Zhang (2017)	1	1	0	1	1	1	1	1	1	1	9	High
8	Amemiya (2019)	0	1	0	1	1	1	1	1	1	1	8	High

Note. 1 = Yes, 0 = No

Q1: Were the aims/objectives of the study clear?

Q2: Was the study design appropriate for the stated aim(s)?

Q3: Was the sample size justified?

Q4: Was the target/reference population clearly defined? (Is it clear who the research was about?)

Q5: Were the risk factor and outcome variables measured correctly using instruments/measurements that had been trialled, piloted or published previously?

Q6: Were the methods (including statistical methods) sufficiently described to enable them to be repeated?

Q7: Were the results presented for all the analyses described in the methods?

Q8: Were the authors' discussions and conclusions justified by the results?

Q9: Were the limitations of the study discussed?

Q10: Was ethical approval or consent of participants attained?

Table S3. Qualitative Research.

**Study #**	**First author (year)**	**Q1**	**Q2**	**Q3**	**Q4**	**Q5**	**Q6**	**Q7**	**Q8**	**Q9**	**Q10**	**Total (%)**	**Quality**
9	Furrer (2015)	1	1	1	1	1	0	0	1	0	1	7 (70%)	Moderate
10	Jouper (2013)	1	1	1	1	1	0	0	1	1	1	8 (80%)	High

Note. 1 = Yes, 0 = No/Unclear/Not applicable

Q1: Is there congruity between the stated philosophical perspective and the research methodology?

Q2: Is there congruity between the research methodology and the research question or objectives?

Q3: Is there congruity between the research methodology and the methods used to collect data?

Q4: Is there congruity between the research methodology and the representation and analysis of data?

Q5: Is there congruence between the research methodology and the interpretation of results?

Q6: Is there a statement locating the researcher culturally or theoretically?

Q7: Is the influence of the researcher on the research, and vice-versa, addressed?

Q8: Are participants, and their voices, adequately represented?

Q9: Is the research ethical according to current criteria or, for recent studies, and is there evidence of ethical approval by an appropriate body?

Q10: Do the conclusions drawn in the research report flow from the analysis, or interpretation, of the data?

© 2019 by the authors. Submitted for possible open access publication under the terms and conditions of the Creative Commons Attribution (CC BY) license (http://creativecommons.org/licenses/by/4.0/).
